# The Complex Interplay of Parasites, Their Hosts, and Circadian Clocks

**DOI:** 10.3389/fcimb.2019.00425

**Published:** 2019-12-12

**Authors:** Priscilla Carvalho Cabral, Martin Olivier, Nicolas Cermakian

**Affiliations:** ^1^Laboratory of Molecular Chronobiology, Douglas Mental Health University Institute, McGill University, Montreal, QC, Canada; ^2^Laboratory of Infectious Diseases and Immunity, Department of Medicine, Research Institute of the McGill University Health Center, McGill University, Montreal, QC, Canada

**Keywords:** parasite, host, circadian rhythms, circadian clock, infection, immune response, behavior

## Abstract

Parasites have evolved various mechanisms to favor infection of their hosts and enhance the success of the infection. In this respect, time-of-day effects were found during the course of parasitic infections, which can be caused or controlled by circadian rhythms in the physiology of their vertebrate hosts. These include circadian clock-controlled rhythms in metabolism and in immune responses. Conversely, parasites can also modulate their hosts' behavioral and cellular rhythms. Lastly, parasites themselves were in some cases shown to possess their own circadian clock mechanisms, which can influence their capacity to infect their hosts. A better knowledge of the circadian regulation of host-parasite interactions will help in designing new preventive and therapeutic strategies for parasitic diseases.

## Introduction

Various aspects of physiology and cell function present self-sustained ~24 h variations termed *circadian rhythms*. In mammals, a central clock located in the Suprachiasmatic Nucleus (SCN) in the hypothalamus controls daily cycles of rest and activity, body temperature, and hormone levels. The discovery of clock genes, encoding the molecular components of circadian clocks (Duguay and Cermakian, 2009; Takahashi, 2017), opened new doors in chronobiology research in challenging this centralized view of the circadian system. First, the rhythmic expression of these clock genes in almost every cell of the body showed that circadian oscillators are virtually ubiquitous (Takahashi, 2017). Second, mouse models with mutated clock genes allowed to investigate the impacts of clock genetic dysfunction on physiology, and tissue-specific roles of circadian clocks (Duguay and Cermakian, 2009).

**Figure 1:**
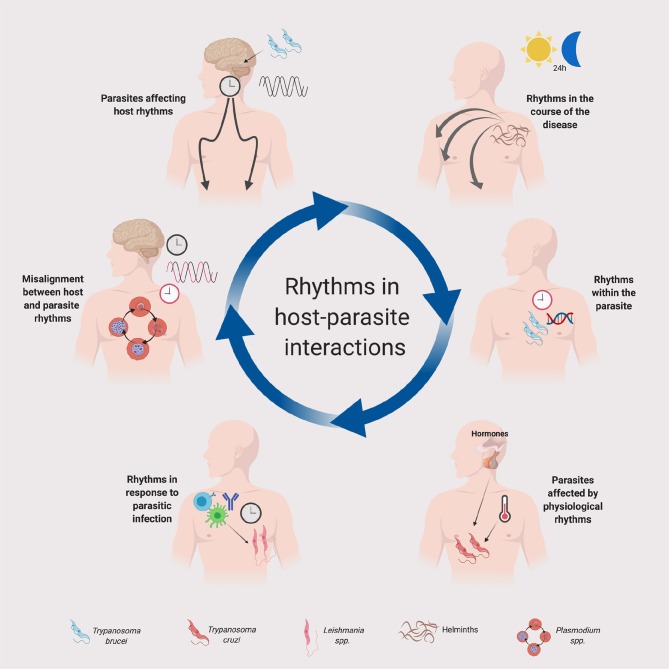
Rhythms in host-parasite interactions. A schematic summary is provided for the implication of daily or circadian rhythms in parasites and their hosts. Host clocks and parasite clocks are depicted in black and red, respectively. The legend at the bottom indicates the parasites represented in the figure. Although in the figure we depicted some specific parasites for illustration purposes, the principles represented in this figure could apply to other parasite groups and species. See text for details. Created with Biorender.com.

Parasitic infections are a major concern worldwide, with millions dying each year and many more living with complications. Curiously, despite an emerging literature about the circadian control of host-microbe interactions, including bacterial and viral infection, very little has been published on the circadian influences on parasitic infections. Various physiological system that could impact parasite life cycles are under circadian control, for example metabolism (Bass, 2012) and immunity (Labrecque and Cermakian, 2015; Nobis et al., 2018). Host physiology could use circadian clocks to anticipate the time of parasite infection and thus optimize its cellular defenses; on the other hand, the parasite itself might have evolved to take advantage of the rhythmicity in the host to enhance its infectivity. This article will first provide an overview of how the host's circadian rhythms can impact parasitic infections. We will then review known circadian rhythms in parasites, as well as parasite-induced changes in the host's circadian rhythms (Figure 1). We refer the readers to recent reviews for discussion of the circadian rhythmicity in insect vectors (Meireles-Filho and Kyriacou, 2013; Rund et al., 2016) and of evolutionary and ecological aspects of daily rhythms in host-parasite interactions (Martinez-Bakker and Helm, 2015; Westwood et al., 2019).

## Rhythms in the Course of the Disease

Clinical and experimental observations have highlighted rhythms in symptoms resulting from parasitic infection (Table 1). For example, patients infected with the malaria parasite *Plasmodium spp*. show oscillations in fever and chills with periods that are multiples of 24 h (Hawking et al., 1968; Karunaweera et al., 1992). This is paralleled by a 24, 48, or 72 h developmental cycle within red blood cells (RBCs), depending on the parasite species (Reece et al., 2017). Indications of rhythms also exist in leishmaniasis: blood samples collected from patients in the daytime contained fewer *Leishmania* amastigotes than night time-collected samples (Saran et al., 1997). In the case of urinary schistosomiasis, a rhythm in egg elimination was observed to peak around midday (Doehring et al., 1985).

**Table 1:** Circadian rhythms in host-parasite interactions for selected parasites^a^.

**Parasite**	**Rhythms in the course of infection**	**Effects of host's immune cells or mediators**	**Parasite-intrinsic rhythms**	**Modulation of host's rhythms**
*Plasmodium* spp.	✓	✓	✓	✓^b^
*Trypanosoma brucei*			✓	✓
*Leishmania* spp.	✓	✓		
Helminths^c^	✓	✓		✓^d^
*Botrytis cinerea*	✓		✓	

a *Check symbols (✓) indicate cases where experimental observations have been made. Absence of check symbol means that to our knowledge, it has not been investigated. See main text for references*.

b *Likely due to an effect of inflammatory mediators*.

c *On this line, observations for various parasitic worms are aggregated*.

d *Only observed at the behavioral level; no evidence yet that the effect is on host's clocks*.

Daily oscillations of the number of microfilariae, an early stage in nematode blood parasite development, have been widely reported. For instance, high nightly counts of *Dirofilaria repens* microfilaria were observed in dogs (Di Cesare et al., 2013). A similar pattern was observed with *Wuchereria bancrofti* (Hawking, 1967), presumably an adaptation to the night-biting *Anopheles* and *Culex* insect vectors (Reece et al., 2017). In contrast, the Pacific-type *W. bancrofti* has a greater number of parasites in circulation in the afternoon (Hawking, 1967), consistent with the phase of biting behavior of its diurnal vector, *Aedes*. Other studies showed that cutaneous abundance of microfilariae in dogs also varied in a manner consistent with the time of peak activity of their vectors (Duke et al., 1967; Otranto et al., 2013). The 24 h rhythm in parasite load in the blood is a consequence of the microfilaria migration to peripheral tissues (Hawking, 1967).

Hawking proposed that parasites benefit from adapting their time of maximal transmissibility to the time of day the insect vector is foraging, and thus, is most likely to take up the parasites (Hawking, 1967; Reece et al., 2017). This was supported by his work on microfilaria described above. Although Hawking proposed that the same could apply to malaria (Hawking, 1970), definitive proof of this has been lacking. In a recent study, however, partial support for the hypothesis was provided using *P. chabaudi*-infected mice: although fewer gametocytes were in circulation at night, and mosquitos were less likely to be infected, nighttime gametocytes were more infectious, as seen by a higher oocyte burden in mosquitos 8 days after blood meal (Schneider et al., 2018).

## Parasites Affected by Physiological Rhythms

### Temperature

The rhythms observed during the course of parasitic infections might be due to host rhythms, such as the body temperature rhythm. Hawking and colleagues addressed this for both microfilaria and *Plasmodium* infections. In microfilaria-infected patients and animals, modifying the body temperature cycles affected microfilaria blood counts (Hawking et al., 1967, 1981). In monkeys infected with *P. knowlesi*, and in chick eggs infected with *P. lophuræ*, an artificial inversion of the body temperature cycle led to a phase shift of the parasite cycles (Hawking et al., 1968). While interesting, these experiments were often on small numbers of animals or patients, so the effects of temperature on parasite rhythms has remained an open question. In experiments where *Plasmodium-*infected mice were kept on an inversed feeding regimen, the body temperature rhythm was unchanged but the intra-RBC development cycle was reversed, showing that the former is not synchronizing parasite development in RBCs (Prior et al., 2018). However, *Trypanosoma brucei* cultured *in vitro* showed rhythms of gene expression that were entrained by temperature cycles (Rijo-Ferreira et al., 2017).

### Melatonin

Melatonin is a well-known circadian hormone in many vertebrates, and several studies addressed its possible role in regulating parasite rhythms. For example, exogenous melatonin administration suppressed the capacity of *Trypanosoma cruzi* epimastigotes to transform into metacyclic forms (Macias et al., 1999). Melatonin was also shown to increase RBC invasion by *Plasmodium chabaudi*, and to affect the maturation of the parasite (Hotta et al., 2000). Effects on parasite maturation were also seen with *P. falciparum* (Beraldo and Garcia, 2005), although no effects of melatonin were found for *P. berghei* and *P. yoelli*, which typically develop asynchronously (Bagnaresi et al., 2009). Furthermore, studies in mice that make low or undetectable levels of melatonin also showed a normal parasite development cycle in RBCs (Hirako et al., 2018; Kennaway, 2019). Thus, even if melatonin plays a role in *Plasmodium* rhythms, it is not necessary and other synchronizers must be involved. As for *Leishmania*, footpad lesions in hamsters following *L. amazonensis* infection were larger when the animals were treated with a melatonin receptor antagonist, and reduced after melatonin treatment (Laranjeira-Silva et al., 2015).

### Feeding and Metabolism

The feeding rhythms of the host can also constitute a timing cue for parasites, as was shown for *P. chabaudi* infection. Two recent studies showed that the parasite stage rhythms in RBCs had a different phase when infected mice were fed during the day or at night (Hirako et al., 2018; Prior et al., 2018). Since the glucose rhythm was shifted in day vs. night-fed mice, it was suggested that it might mediate the effect on the parasites (Prior et al., 2018). Cytokines seem to be involved in this glucose regulation during infection, and rhythms in parasite developmental stages were abolished in IFNγ KO mice, in TNFα receptor mice, and in mice lacking IFNγ receptor in hematopoietic cells (Hirako et al., 2018). Similar results were found in chemically-induced diabetic mice, which have constant high glucose blood levels (Hirako et al., 2018). TNFα involvement is reminiscent of the dynamic relationship between rising blood TNFα levels preceding body temperature increases in patients infected with *P. vivax* (Karunaweera et al., 1992).

## The Impact of Immune Rhythms on the Response to Parasitic Infections

All cells of the immune system express clock genes, and immune cells such as macrophages and T cells have 4–8% of their transcriptome under circadian regulation (Keller et al., 2009; Nobis et al., 2019). Accordingly, a role was found for circadian clocks in regulating various immune responses, including during infection (Nobis et al., 2018). For example, various functions of macrophages and monocytes, including phagocytosis, secretion of cytokines and trafficking between tissues, are clock-controlled (Labrecque and Cermakian, 2015; Nobis et al., 2018). A rhythm was found in the expression of many of the pattern recognition receptors (PRRs), as well as in key signaling molecules downstream of these receptors, involved in responses to pathogen-associated molecular patterns (PAMPs) (Nobis et al., 2018; Silver et al., 2018). Rhythms in chemokines and their receptors can also lead to the time-dependent recruitment of immune cells to an infection site. Therefore, both the numbers of immune cells and their responsiveness to signals can vary according to the time of day. Given the interplay between parasites and immune cells (in particular those serving as hosts for the parasites) (Gazzinelli et al., 2014; Atayde et al., 2016), an influence of the immune rhythms on the progression of parasitic disease is likely. Such a circadian influence was indeed uncovered in a few recent studies.

The infection of mice by *Leishmania* parasites during late day led to larger lesions than upon infection in the late night (Laranjeira-Silva et al., 2015), and this is likely due to circadian regulation because similar rhythms were seen in *L. major*-infected mice kept in constant darkness (Kiessling et al., 2017). Interestingly, late day/early night infection led to a stronger recruitment of innate immune cells such as neutrophils and macrophages, which serve as host cells for *Leishmania*. The rhythms in parasite load, immune cell recruitment and chemokine expression were abolished in mice lacking a circadian clock specifically in cells of hematopoietic origin, demonstrating a role of immune cell clocks in regulating *Leishmania* infection (Kiessling et al., 2017).

A circadian regulation was also seen in the case of infection with the helminth *Trichuris muris*. While the *Leishmania* study looked at the immediate inflammatory response, the time of *Trichuris* infection led to a difference in the timing of worm expulsion and antibody production a few weeks later (Hopwood et al., 2018). Knocking out clock function specifically in dendritic cells led to a loss of the time-dependency (Hopwood et al., 2018).

## Evidence for Circadian Timing Mechanisms Within Parasites

Another layer of complexity is the possibility that the parasites themselves might have their own endogenous clock (Table 1). This is likely, given that various single-cell eukaryotes display endogenous circadian rhythms [e.g., green alga *Ostreococcus tauri* (Pfeuty et al., 2012), dinoflagellate *Lingulodinium polyedra* (Hastings, 2007), fungus *Neurospora crassa* (Dunlap et al., 2007)]. Recently, a study showed endogenous circadian rhythms of gene expression in *Trypanosoma brucei* (Rijo-Ferreira et al., 2017). Isolated parasites were synchronized using temperature cycles and RNA sequencing was performed to identify transcripts varying with a 24 h period. About 15% of all transcripts were found to be rhythmic. The rhythms were temperature compensated, a defining feature of circadian clocks. Pathway analysis showed that many of the oscillating transcripts were involved in metabolic functions. Interestingly, the sensitivity of the parasite to oxidative stress and its resistance to the drug suramin were both under circadian control, underscoring the biological significance of parasite-borne rhythms (Rijo-Ferreira et al., 2017).

A recent report has addressed a similar question in *Plasmodium* (Subudhi et al., 2019). Mice infected with *P. chabaudi* (which displays a 24 h development cycle in RBCs) were housed under a light-dark cycle, and blood sampled over 30 h was used for RNA sequencing: over 5,000 *P. chabaudi* transcripts displayed ~24 h rhythms. However, since these rhythms could be driven by host signals, published transcriptomic data from *in vitro*-cultured *P. falciparum* were reanalyzed for ~24 h period variation. The *P. falciparum* development cycle period is 48 h, allowing transcripts dependent on this cycle to be distinguished from circadian transcripts. A significant 24 h component was found for ~500 transcripts. Among these, 110 genes were also among the rhythmic *P. chabaudi* genes, suggesting they were endogenously generated in *P. chabaudi* as well.

Although the identification of endogenous timing mechanisms in protozoan parasites is in its infancy and presents some challenges, the situation can be different for other groups of parasites. Indeed, research on fungal parasites might benefit from the detailed knowledge about the molecular clock of the fungus *Neurospora crassa*. For example, a study on the plant pathogen *Botrytis cinerea* described the existence of genes homologous to clock components White Collar Complex and Frequency (FRQ) of *N. crassa* (Hevia et al., 2015; Larrondo and Canessa, 2019). These *B. cinerea* clock genes function in an analogous way, with a core feedback loop where FRQ negatively feeds back on its own expression.

As for helminths, it is likely that like other animals they have a molecular clock machinery. Although helminths are not a monophyletic group, knowledge about clock mechanisms in the nematode *Caenorhabditis elegans* [for which circadian research is beginning (Goya et al., 2016; Olmedo et al., 2017)] might be of help.

## Misalignment of Host-Parasite Rhythms

Many parasites display circadian or daily rhythms on their own, either generated by endogenous clocks or conferred by a rhythmically active vector. A consequence is that the alignment of these parasite rhythms with those of the vertebrate host might be beneficial for either the parasite or the host, and conversely, that the misalignment of the host and parasite rhythms might impact the host-parasite relationship. A few studies have directly tackled this question.

The Reece group has taken advantage of the 24 h rhythms in the intra-RBC development cycle of *P. chabaudi* to determine the impact of a mismatch between rhythms in the host and parasites. Infected RBCs were used to infect recipient mice which were either on the same light-dark cycle as the donor mice, or an inverted light-dark cycle. A mismatch between parasite and host rhythms led to reduced parasite density and gametocyte production (O'Donnell et al., 2011), confirming that host-parasite rhythm alignment aids the parasite. A follow-up study showed that this mismatch effect does not depend on either the developmental stage of the parasite used for infection or on the route of infection, and has an impact at early infection stages (O'Donnell et al., 2013). A misalignment of the *P. chabaudi* and host rhythms also impacted rhythmic parasite genes, and led to a reduced development cycle period (Subudhi et al., 2019).

The existence of a circadian clock in the fungus *B. cinerea* (see above) has allowed researchers to investigate the interplay between the clocks in the pathogen and in the infected plant (Larrondo and Canessa, 2019). The outcome of the infection of *Arabidopsis thaliana* by *B. cinerea* depends on the time of day at which it occurs (larger infected area at dusk; Hevia et al., 2015). Experiments using clock gene mutants of either *B. cinerea* or *A. thaliana* showed that the clock in the fungus is necessary for this time-dependency. Moreover, in experiments where the two organisms' clocks were entrained on out-of-phase light-dark cycles, dusk fungus always induced larger lesions, independent of the plants phase (even though it had previously been shown that plants have more efficient defenses against necrotrophic fungi in the morning; Hevia et al., 2015).

## Effect of Parasites on the Host's Circadian Rhythms

### Effects on Behavioral Rhythms

Many examples of parasites affecting the host's daily behavior are known (Table 1). In particular, parasites can manipulate an intermediate host to optimize transmission to the final (vertebrate) host, thus completing their life cycle (Lefevre et al., 2009; de Bekker et al., 2014). Here we review some examples where host's behavior manipulation has a daily component.

Snails *Potamopyrgus antipodarum* are the intermediate hosts for the trematode parasitic worm *Microphallus*. Snails carrying the infective forms of the parasite were found on the top of rocks in the morning, at a time where the ducks (final hosts for the parasite) are feeding, but later in the morning and for the rest of the day, were underside the rocks to escape predation by fish (not final hosts for *Microphallus*; Levri and Lively, 1996). This is consistent with manipulation of *P. antipodarum* by the parasite, although it could rather be that increased hunger of the snails extends their foraging duration. Another trematode using a snail as an intermediate host, *Schistosoma mansoni*, shows a daily rhythm of emergence of the larval stage that transfers to the final mammalian host. Interestingly, emergence occurs in the daytime for parasites infecting humans (diurnal) and in the evening for parasites infecting rats (nocturnal) (Mouahid et al., 2012).

The case of insect-manipulating fungi is also interesting. One striking example is the manipulation of ants (*Camponotus leonardi*) by parasitic fungus *Ophiocordyceps unilateralis*. Infected ants abandon their normal activities and climb to the top of plants before biting leaves and dying (de Bekker et al., 2014). This favors the dissemination of the parasite's spores. Interestingly, this behavioral manipulation is highly synchronized, with “zombie” ants exhibiting the biting behavior around midday (Hughes et al., 2011). Recent transcriptomic analysis of *O. unilateralis* has uncovered many rhythmic transcripts in the fungi, including putative clock components, which could be involved in the daily regulation of the infection and host manipulation (de Bekker et al., 2017).

### Effects on Circadian Clocks

Human African trypanosomiasis (sleeping sickness) is a parasitic disease that perfectly illustrates the capacity of parasites to modulate host's rhythms. Trypanosomiasis is caused by the protozoan parasite *Trypanosoma brucei*, transmitted by tsetse fly's bite. Despite the common name of the disease (which comes from the coma observed in patients where the parasite has invaded the brain), infected patients do not show hypersomnia, but a disorganization of their sleep-wake cycles, becoming worse as the disease progresses (Buguet et al., 1993). An advance of the melatonin rhythm was also observed, but no change in core body temperature rhythm (Claustrat et al., 1998). In *T. brucei*-infected rats, however, both a phase shift of the temperature rhythm and changes in sleep profile were observed (Grassi-Zucconi et al., 1995; Seke Etet et al., 2012). Interestingly, some of the phenotypes were present even before parasites start accumulating in the brain, although they became more prominent after that stage (Seke Etet et al., 2012). Disorganized sleep-wake cycles and a phase advance of rhythms controlled by the SCN central clock begs the question of whether the infection causes changes of circadian clocks themselves. A number of studies have addressed the effects of *T. brucei* infection on SCN function. In infected rats, a reduced firing rate of SCN neuron and a phase-advance of their activity rhythms was observed (Lundkvist et al., 1998), as well as disrupted synaptic activity (Lundkvist et al., 2002), and an altered Fos expression profile (Bentivoglio et al., 1994). Moreover, infection led to a reduced expression of glutamate receptors, which mediate light response, and consistently, to a reduced response of the clock to light (Peng et al., 1994).

An in-depth analysis of *T. brucei*'s ability to disrupt host rhythms was recently published (Rijo-Ferreira et al., 2018). *T. brucei*-infected mice showed increased daytime activity and a shifted temperature rhythm under a light-dark cycle, and a shorter free-running period. Further analysis revealed that an advance of the endogenous rhythms seemed to underlie the daytime activity. Interestingly, the short period phenotype was observable before the occurrence of the parasite in the brain, suggesting an indirect effect via a peripheral factor. Tissues of infected (and control) PER2::Luciferase mice were put in culture, showing a shortened period and a phase advance of the rhythms in some peripheral tissues, including adipose tissue, an organ with high parasitemia. Such effects were blunted upon suramin treatment. Similar effects were also found in tissues infected *in vitro*, excluding the involvement of systemic cues, for example due to inflammation (Rijo-Ferreira et al., 2018). The *in vitro* data is consistent with a prior report of a shortened period in the pituitary, but not the SCN, of infected animals (Lundkvist et al., 2010) after short term infection (in the Rijo-Ferreira study, an effect on the SCN was seen only after long-term infection).

The multimammate mouse, *Mastomys natalensis*, was used as a rodent model susceptible to both the human parasite *T. brucei gambiense* and *T. brucei brucei*, a pathogen not infectious to humans. Interestingly, infection with *T. b. gambiense*, but not with *T. b. brucei*, led to a 30% decrease in SCN neuronal density, while astrocyte activation was noted for both (Tesoriero et al., 2018). This, together with the phase shift of melatonin rhythm in human patients (Claustrat et al., 1998), suggests that the effects on host's circadian clocks also exist in human patients.

Despite all these reports on *T. brucei*, very little is known on the effect of other parasites on host clock mechanisms. *Trypanosoma cruzi*-infected mice showed a lower amplitude of locomotor activity rhythms, attenuated light responses and slower entrainment to a new LD cycle (Fernandez Alfonso et al., 2003). Mice infected with *Plasmodium chabaudi* displayed reduced locomotor activity and body temperature at night, in a parasite genotype-specific way (Prior et al., 2019). Such effects might be due to the inflammation occurring in the host, as various immune mediators were shown to affect circadian rhythms (Cermakian et al., 2014). This is supported by the work of Rijo-Ferreira et al.: in parallel to *T. brucei*, they tested behavioral and bioluminescence rhythms in mice infected with *P. chabaudi*. A suppression of activity levels and clock gene expression in adipose tissue was found, both likely acute effects of systemic inflammation, as no effects were seen on the period of rhythms in cultured tissues (Rijo-Ferreira et al., 2018).

Knowledge of the effect of *Leishmania* infection on host clocks is even scarcer: the only study that has addressed this question showed that *L. amazonensis* does not alter melatonin levels or rhythms in hamsters up to 21 days post infection (Laranjeira-Silva et al., 2015). A manipulation of clock mechanisms may also occur in fish: in a transcriptomic analysis of Nile tilapia infected with *Saprolegna* parasite vs. non infected controls, one of the most prominent category of modified genes was that of clock genes and regulators (Ellison et al., 2018).

## Concluding Remarks

There are now many examples of the importance of circadian or daily rhythms in host-parasite interactions. So far, data has been mostly descriptive, and future research will need to address in particular: (1) how host circadian clocks and rhythms impact the initiation and outcome of parasitic infections; (2) the mechanisms involved in the effects of the parasites on host behavior and cellular functions; (3) the parasite-intrinsic rhythms and the clock mechanisms that underlie them.

Daily rhythms can represent an adaptative mechanism used by parasites to favor their transmission, e.g., to become available to be taken up by an intermediate vector or by the final vertebrate host. They can also enhance parasite survival by aligning parasite activity with the rhythmic metabolism, immunity, and behavior of the host, as well as environmental cycles in abiotic environments. Therefore, a better knowledge of the mechanisms underlying the circadian regulation of host-parasite interactions may allow the design of new strategies to prevent and control parasitic infections. It will also be beneficial to extend this research beyond more common parasites such as *Plasmodium* and *T. brucei*, to neglected tropical diseases such as filariasis, which are known to have a clear time-of-day regulation and affect hundreds of million people worldwide.

## Author Contributions

PC, MO, and NC wrote the paper.

### Conflict of Interest

The authors declare that the research was conducted in the absence of any commercial or financial relationships that could be construed as a potential conflict of interest.
